# Case report: Identification of Hepatitis B Virus in the cerebrospinal fluid of neuromyelitis optica spectrum disorders and successful treatment with ofatumumab and inebilizumab

**DOI:** 10.3389/fimmu.2024.1351782

**Published:** 2024-02-15

**Authors:** Linjun Cai, Xu Liu, Hongyu Zhou, Jinmei Li, Dong Zhou, Zhen Hong

**Affiliations:** ^1^ Department of Neurology, West China Hospital of Sichuan University, Chengdu, Sichuan, China; ^2^ Institute of Brain Science and Brain-inspired Technology of West China Hospital, Sichuan University, Chengdu, Sichuan, China; ^3^ Department of Neurology, Chengdu Shangjin Nanfu Hospital, Chengdu, Sichuan, China

**Keywords:** neuromyelitis optica spectrum disorders, hepatitis B virus, ofatumumab, inebilizumab, case report

## Abstract

Neuromyelitis optica spectrum disorder (NMOSD) is a rare demyelinating disease of the central nervous system primarily affecting the optic nerves, spinal cord, and brainstem. Viral infection may trigger NMOSD. Here, we report the case of a 34-year-old female presenting with a range of symptoms including nausea, vomiting, dysphagia, choking, and fatigue with unsteady gait, diplopia, hearing loss, left-sided facial paralysis, breathing difficulties, and hoarseness of voice. Her HBV DNA concentration, as determined by quantitative PCR analysis, exceeded 5×10^7^ IU/ml in serum and 4.48×10^2^ IU/ml in CSF. Next-generation sequencing of CSF revealed 1,528 HBV sequences in DNA analysis and 6 sequences in RNA analysis. Serum aquaporin-4 antibody (AQP4-Ab) titer was 1:10, and the CSF titer was 1:3.2. Brain magnetic resonance imaging showed high signal intensities in the brain stem, medulla oblongata, and left middle cerebellar peduncle with mild restricted-diffusion. The patient received antiviral and hepatoprotective medications before the high-dose methylprednisolone pulse therapy. However, the patient did not respond well to the first-line treatment. Subsequently, the patient received ofatumumab and inebilizumab. Throughout the follow-up period, there was a gradual improvement in her neurological symptoms, with no reactivation of hepatitis B or deterioration of liver function observed. Thereby, to the best of our knowledge, we report the first case of successful treatment with ofatumumab and inebilizumab in a patient with NMOSD concurrent with HBV infection.

## 1 Introduction

Neuromyelitis optica spectrum disorder (NMOSD) is a rare demyelinating disease of the central nervous system (CNS), primarily affecting the optic nerves, spinal cord, and brainstem (1). The current literature, including numerous studies and case reports, suggests an association between infections, vaccinations, and the onset of CNS demyelinating diseases, including NMOSD (2–6).

Chronic hepatitis B virus (HBV) infection is a significant global public health concern, with an estimated 296 million people infected in 2019 (7). The prevalence of HBV varies geographically; for example, China has approximately 70 million carriers of hepatitis B surface antigen (HBsAg), indicating a prevalence of 5%-6% (8, 9). Despite its global impact, NMOSD with active HBV replication remains rare. While many previous case reports have discussed the coexistence of NMOSD and HBV infection (3, 6), direct evidence of HBV presence in cerebrospinal fluid (CSF) is lacking, necessitating further research to establish a conclusive correlation between HBV and NMOSD.

In the context of immunosuppressive therapy for NMOSD, options such as high-dose intravenous methylprednisolone pulse therapy and other immunosuppressants like azathioprine (AZA), mycophenolate mofetil, ofatumumab, and inebilizumab are being used (10). It is crucial to acknowledge that immunosuppressive therapy may disrupt the immunologic control over HBV infection, potentially leading to HBV reactivation (HBVr) (11, 12). Therefore, selecting an appropriate treatment for NMOSD patients with coexisting HBV infection poses substantial challenges.

Despite its clinical importance, a consensus on the optimal approach to treating NMOSD patients with coexisting HBV infection is lacking. This report addresses this gap by presenting the first case of NMOSD with positive aquaporin-4 antibody (AQP4-Ab) and simultaneous detection of HBVr in both serum and CSF. The patient manifested symptoms consisted with area postrema syndrome (APS) and brainstem syndrome (BS). NMOSD remission was achieved through immunotherapy, including high-dose methylprednisolone pulse therapy, ofatumumab, and inebilizumab, without triggering HBVr. Further research is essential to refine the management strategies for NMOSD patients with concurrent HBV infection.

## 2 Case description

In May 2023, a 34-year-old Tibetan female was admitted to our hospital with a constellation of symptoms, including persistent nausea and vomiting for one month; dysphagia and choking for 25 days; and fatigue with unsteady gait for 20 days, which then followed by diplopia, hearing loss, left-sided facial paralysis, breathing difficulties, and voice hoarseness for 5 days. Before the onset of the illness, she experienced mild diarrhea that resolved within one day. The ongoing nausea and vomiting led to poor appetite, mental distress, and fatigue, necessitating intravenous nutritional support. Notably, she also had a past medical history of untreated chronic hepatitis B for 6 years. The patient denied any history of smoking or alcohol consumption, had no psychosocial background, and no family history of genetic diseases. Furthermore, there was no reported exposure to heavy metals.

Before seeking treatment at our hospital, the patient had previously consulted the gastroenterology department in another hospital for nausea and vomiting. Various investigations including routine blood test, amylase, lipase, electrolytes, and liver and kidney function tests were all performed with negative results. Abdominal CT and gastrointestinal endoscopy were also unremarkable. Symptomatic treatments, such as antiemetics and intravenous fluid therapy, were prescribed, but there was no improvement. Consequently, the patient was referred to our hospital for further evaluation.

**Figure 1:**
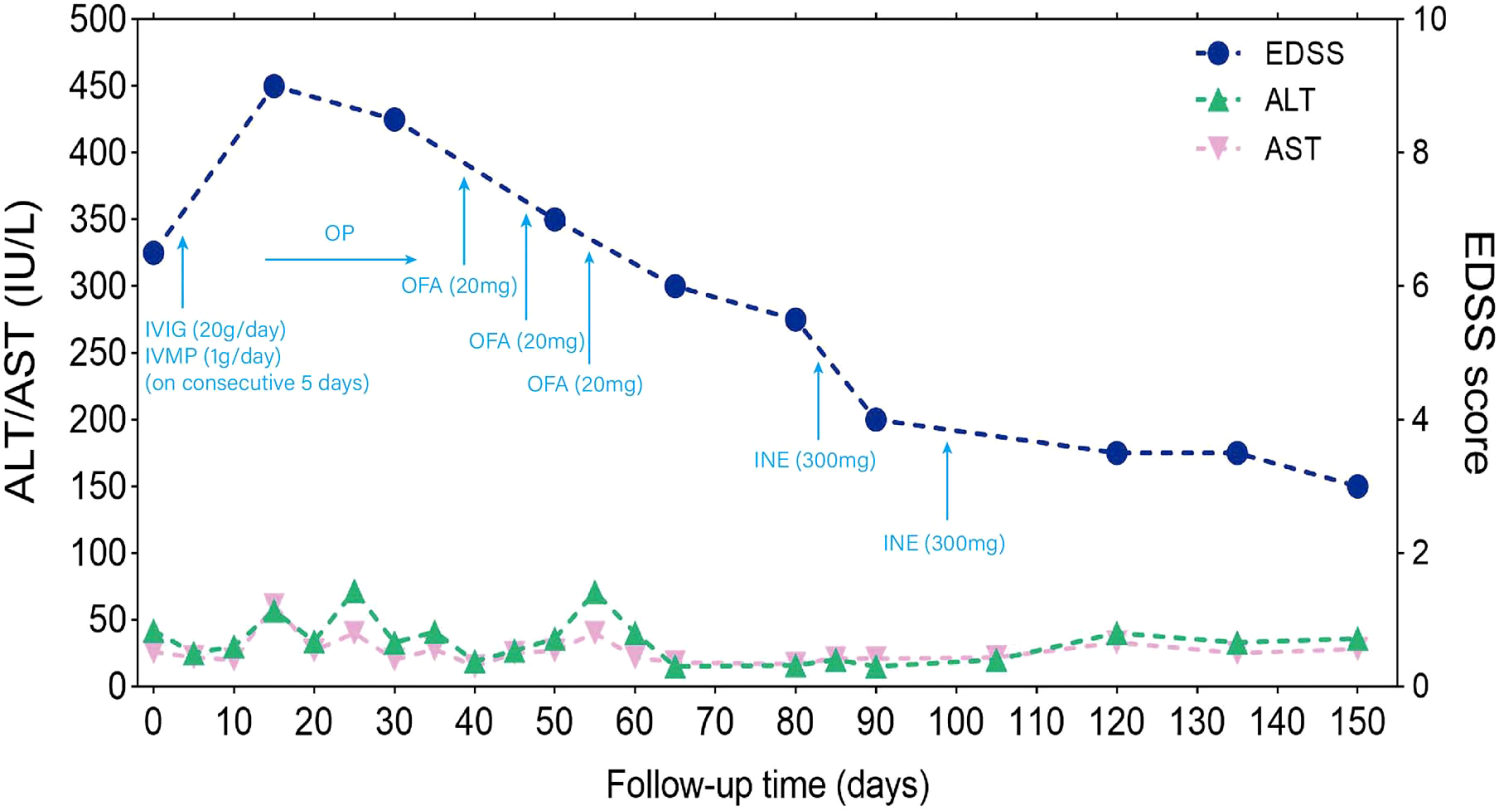
Timeline of disease disability course and different treatment regimes. The x-axis indicates the number of days after admission. The left y-axis indicates ALT (IU/L) and AST (IU/L). The right y-axis also indicated EDSS score. ALT: normal values < 40 IU/L ml; AST: normal values < 35 IU/L; EDSS is used to quantify the level of disability in NMOSD, ranging from 0 to 10, with higher scores representing increasing levels of disability; IVIG: 0.4 g/kg intravenous immunoglobulins daily for 5 days; IVMP, intravenous methylprednisolone 1000 mg for 5 consecutive days, followed by oral prednisone (1 mg/kg) with a weekly reduction of 5 mg. Throughout the entire immunotherapy process, ALT and AST showed some fluctuations without significant elevation. There was no clinical improvement after receiving IVMP and IVIG, and the EDSS score worsened to 9 on day 15 from admission. Ofatumumab was subcutaneously injection on days 39, 46, 53 (20 mg per dose) from admission; inebilizumab was intravenously infusion on days 83, 90 (300 mg per dose) from admission. After receiving ofatumumab and inebilizumab treatments, there was a gradual improvement in symptoms, and the EDSS score changed from 9 to 3. ALT, aspartate aminotransferase; AST, alanine aminotransferase; IVMP, intravenous methylprednisolone; IVIG, intravenous immunoglobulin; OP, oral prednisone; OFA, Ofatumumab; INE, inebilizumab; EDSS, Expanded Disability Status Scale.

Upon admission, the physical examination revealed no abdominal tenderness or rebound pain and no signs of peritoneal irritation. The initial Expanded Disability Status Scale (EDSS) score was 6.5 points (**Figure 1**). The EDSS is a tool utilized to quantify neurological impairment in NMOSD, ranging from 0 to 10, with higher scores indicating more severe disability (13).

## 3 Diagnostic assessment

**Figure 2:**
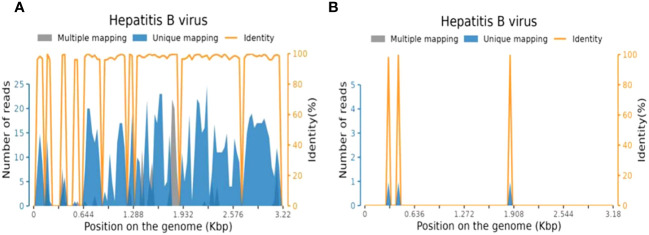
Sequence reads mapped to HBV in CSF from NGS results. **(A)** Sequence reads of HBV DNA in CSF with a total coverage of 69.18% and an average depth of 6.22 X; **(B)** Sequence reads of HBV RNA in CSF with a total coverage of 1.57% and an average depth of 1.00 X. HBV, hepatitis B virus; CSF, cerebrospinal fluid; NGS, next generation sequencing.

The patient’s serum was positive for hepatitis B surface antigen (HBsAg), hepatitis B e antigen (HBeAg), and hepatitis B core antigen (HBcAg). The concentration of HBV DNA in the serum, as determined by quantitative PCR analysis, exceeded 5×10^7^ IU/ml. The HBV DNA concentration in CSF was also found to be greater than 4.48×10^2^ IU/ml. On next-generation sequencing (NGS) of CSF, DNA analysis showed 1,528 HBV sequences, and RNA analysis showed 6 HBV sequences. The sequencing was performed using the Illumina NextSeq 550 sequencing platform (Illumina, San Diego, USA) and a SE75bp sequencing strategy (**Figure 2**). The test for anti-AQP4 antibodies was positive in both the serum (titer, 1:10) and CSF (titer, 1:3.2) with a commercial kit (Euroimmun, Germany) (**Supplementary Figure 1**). Other auxiliary laboratory examinations are listed in **Table 1**.

**Table 1:** Laboratory auxiliary examinations.

Patient	
Sex	female
Age (years)	34
Nation	Tibetan
tumor markers	
Hematologic tests	
Hemoglobin (g/L, normal values between 115 to 150)	167
Tumor markers (CA15-3, CA19-9, CA125, CA72-4, AFP, CEA)	(-)
Thyroid function	(-)
Treponema pallidum antibodies	(-)
Inflammatory factor	
C-reactive protein (mg/L, normal values < 5)	21.3
PCT (ng/mL, normal values < 0.046)	0.06
IL-6 (pg/mL, normal values between 0 to 7)	24.27
Liver function tests	
ALT (IU/L, normal values < 40)	42
AST (IU/L, normal values < 35)	26
Bilirubin (umol/L, normal values between 5 to 28)	25.3
Microbiological tests	
HBsAg (normal values between 0 to 0.08)	>250
HBsAb (normal values between 0 to 10)	<2
HBeAg (normal values between 0 to 0.1)	>200
HBeAb (normal values >1)	15.41
HBcAb (normal values >1)	0.02
HBV-DNA (copies/ml, normal values < 1×10^2^) (serum)	>5×10^7^
Tuberculosis	(-)
Human immunodefciency virus	(-)
TORCH	(-)
Parasites	(-)
Immunological test results	
IgG4	(-)
Rheumatoid factor	(-)
AKA	(-)
ANA	(-)
Anti-dsDNA	(-)
Anti-SM	(-)
Anti-SS-A and SS-B	(-)
Anti-ANCA	(-)
Anti-Scl-70	(-)
Anti-Jo-1	(-)
Anti-RNP	(-)
Anti-ACA	(-)
CSF	
Karyocyte (×10^6^/L, normal values between 0 to 10)	90
Erythrocytes (×10^6^/L)	0
Protein (g/L, normal values between 0.15 to 0.45)	0.26
Smear, culture and ink stain	(-)
Oligoclonal bands (CSF/serum)	(-)
CNS demyelinating antibodies^a^	(CSF/serum)
Anti-AQP4	(1:3.2/1:10)
Anti-MOG	(-/-)
Anti-GFAP	(-/-)
Anti-MBP	(-/-)
Anti-AQP1	(-/-)

*a: cell base assay (Euroimmun, Germany).

AFP, alpha fetoprotein; AKA, anti-keratin antibodies; ALT, alanine aminotransferase; ANA, anti-nuclear antibody; Anti-AQP1, anti-aquaporin-1 antibodies; Anti-AQP4, anti-aquaporin-4 antibodies; Anti-ANCA, anti-Neutrophil Cytoplasmic antibodies; Anti-ACA: anti-cardiolipin antibodies; Anti-dsDNA, double-stranded deoxyribonucleic acid; Anti-GFAP, anti-glial fibrillary acidic protein antibodies; Anti-MBP, anti-myelin basic protein antibodies; Anti-MOG, anti-myelin oligodendrocyte glycoprotein antibodies; Anti- RNP, ribonuclear protein; Anti-SM, anti-Smith antibodies; Anti-SS-A and SS-B, Anti-SS-A(Ro) and anti-SS-B(La) autoantibodies.

AST, aspartate aminotransferase; CNS, central nervous system; CA, cancer antigen; CEA, carcino-embryonic antigen; CSF, cerebrospinal fluid; HBcAb, hepatitis B core antibody; HBeAb, hepatitis B e antibody; HBeAg, hepatitis B e antigen; HBsAb, hepatitis B surface antibody; HBsAg, hepatitis B surface antigen; HBV, hepatitis B virus; HIV, human immunodeficiency virus; PCT, Procalcitonin; TORCH, toxoplasma, treponema pallidum, rubella virus, cytomegalovirus, and herpes simplex virus 1 and 2.

**Figure 3:**
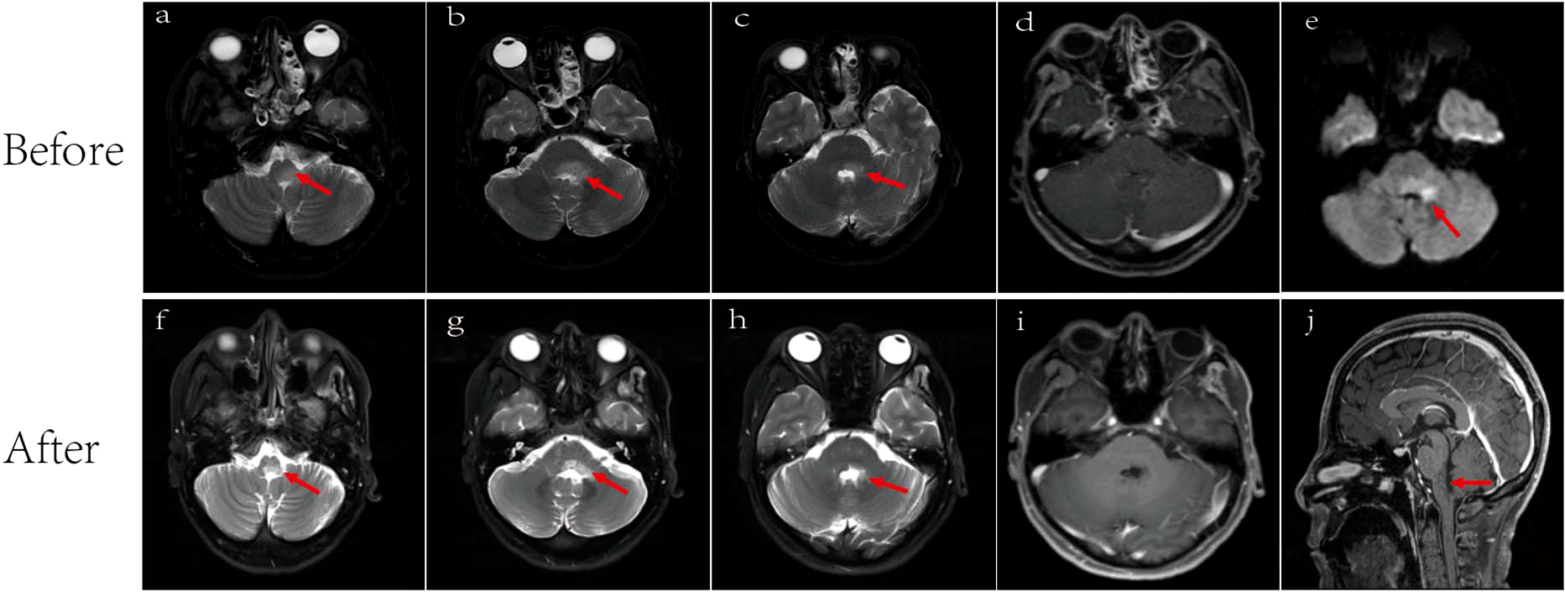
Brain MRI. Lesions (arrowed) on brain MRI images before **(A–E)** and after **(F-J)** antiviral and immunotherapy showing obviously manifested shrinkage after treatment. **(A)** T2-weighted showed medulla oblongata lesion. Lesion area: 2.4 cm^2^; **(B)** T2-weighted showed dorsal aspect of the brainstem and left cerebellar peduncle lesions. Lesion area: 3.3 cm^2^; **(C)** T2-weighted showed dorsal aspect of the brainstem and left cerebellar peduncle lesions. Lesion area: 1.8cm^2;^ **(D)** No significant enhancement before treatment; **(E)** Diffusion-weighted MRI (DWI) showed mild limited diffusion. Lesion area: 1.7 cm^2^; **(F)** T2-weighted showed a reduction in the area of medullary lesions, with an area of 1.0 cm²; **(G)** T2-weighted showed a reduction in the area of brainstem and left cerebellar peduncle lesions, with an area of 2.0 cm^2^; **(H)** T2-weighted showed a reduction in the area of brainstem and left cerebellar peduncle lesions, with an area of 0.7 cm^2^; **(I)** No significant enhancement after treatment; **(J)** Sagittal view showed medulla oblongata lesion.

Additionally, comprehensive investigations, including routine stool analysis, abdominal ultrasound, enhanced CT scans of the upper and lower abdomen, gastroscopy, upper abdominal MRI, and tests for pancreatic enzymes, lipase, and amylase all revealed no abnormalities, thereby ruling out digestive system abnormalities. EEG result was within normal limits. Brain MRI using T2-weighted sequences showed high signals in the brain stem, medulla oblongata, and left middle cerebellar peduncle with mild restricted-diffusion, but no significant enhancement (**Figures 3A–E**).

According to the 2015 International Diagnostic Consensus for NMOSD (1), the patient’s clinical symptoms and MRI findings align with two (APS and BS) of the six core clinical syndromes of NMOSD. The comprehensive gastrointestinal examination ruled out digestive system diseases. CSF analysis and the results of other antibodies targeting specific CNS demyelinating diseases, such as anti-MOG and anti-GFAP antibodies, also ruled out other CNS infections and demyelinating diseases. Whereas the presence of positive APQ4-Ab in both serum and CSF confirms the diagnosis of NMOSD.

## 4 Therapeutic process and follow-up

During the 150-day follow-up period, the timeline of disease disability, different treatment regimens, and changes in liver function were depicted in **Figure 1**. Upon admission, an aggressive treatment approach was initiated, including intravenous high-dose methylprednisolone (1g/day) and immunoglobulins (0.4mg/kg) for five days, followed by a tapered oral prednisone regimen (1mg/kg) with a weekly reduction of 5mg. To inhibit HBV replication, entecavir (0.5mg/day) was prescribed, alongside propofol tenofovir fumarate tablets and hepatoprotective drugs. It is noteworthy that although plasmapheresis (PE) is also considered a first-line treatment for NMOSD, we opted not to go for PE in this patient because he already had received IVIG, and the use of PE may attenuate the effect of IVIG.

On day 15, the patient developed steroid-related complications including gastrointestinal bleeding and severe pulmonary infection accompanied by a decrease in oxygen saturation. The EDSS score worsened to 9. Therefore, immediate interventions including non-invasive ventilator support, antimicrobial therapy, gastric acid suppression, and intravenous nutritional support, among other symptomatic treatments were implemented. After implementing these interventions along with a gradual reduction in oral prednisone, a repeated chest CT on day 38 showed a decrease in pulmonary inflammation as compared with the previous scan. The inflammatory markers also significantly decreased and her temperature returned to the baseline. ALT and AST levels were relatively stable. Hemoglobin and stool occult blood were normal, with no signs of gastrointestinal bleeding. At this point, immunotherapy was introduced, with subcutaneous ofatumumab injections on days 39, 46, and 53 (20 mg per dose) from admission.

After the administration of ofatumumab, the patient’s symptoms gradually improved (**Figure 1**), and no HBVr was observed. We then considered switching to inebilizumab, a humanized monoclonal antibody targeting CD19-positive B cells, that was approved by the Food and Drug Administration (FDA) in 2020 for relapse prevention in adult patients with AQP4-Ab seropositive NMOSD. Therefore, the patients received inebilizumab infusion (300 mg) on days 83 and 98.

The patient demonstrated good compliance with the recommended intervention and actively participated in follow-up assessments, which included liver and kidney function tests, complete blood count, electrolyte levels, and CSF examination. All test results were within normal limits. By day 90, significant progress was evident, with brain MRI revealing reduced lesions compared to the previous scan (**Figures 3F–J**) and the HBV DNA in serum dropping to 1.32×10^5^ IU/mL. The patient’s symptoms also improved, reflected in the EDSS score, which decreased from 9 to 3 (**Figure 1**). Interestingly, the treatment was well-tolerated, with no hepatitis outbreaks, deterioration of liver function, or any adverse reactions reported. Additionally, the count of CD20-positive B cells decreased from 238 cells/ul to 5 cells/ul, indicating a positive response to therapy.

## 5 Discussion

This is the first report of detectable HBV in the CSF of a patient with NMOSD who was successfully treated with ofatumumab and inebilizumab. NMOSD is a rare autoimmune disorder characterized by recurrent optic neuritis (ON) and transverse myelitis (TM). While the exact etiology remains elusive, substantial evidence implicates infectious agents, primarily viral infections (3, 6, 14). The 2015 international diagnostic consensus for NMOSD recognizes six major clinical characteristics including APS and BS (1). Notably, the area postrema, a vomiting center in the caudal and dorsal brainstem, lacks a blood-brain barrier and exhibits heightened expression of AQP4-Ab. The involvement of this area potentially leads to intractable nausea, vomiting, or hiccups (15). Brainstem syndromes, overlapping with area postrema syndrome, may also manifest with oculomotor dysfunction (e.g., diplopia and nystagmus) or other cranial nerve palsies (16). In this report, our patient presented with APS and BS. In the previously reported case series, ON and TM were the most frequently observed clinical characteristics among NMOSD patients with concurrent HBV infection; but one case presented with BS (3, 6, 14). The current literature suggests that ON tends to be more aggressive among people with chronic HBV infection compared to those without HBV (17). Similarly, our patient exhibited significant disability at disease onset. Further research is warranted to explore the relationship between HBV load in the CSF and the severity of NMOSD.

Numerous studies have noticed a relationship between viral infections and the development of NMOSD (2–6), with some having proposed a role for Helicobacter pylori and Clostridium perfringens (18, 19). By using NGS testing, we detected HBV DNA and HBV RNA in the CSF and ruled out other infections. Despite the decrease in HBV replication activity with the administration of anti-HBV drugs and hepatoprotective agents, HBV DNA was still detectable in the CSF even after treatment. A recent study suggested that HBV DNA can persist in the CSF even after antiviral therapy, this is possibly due to the limited penetration of most nucleos(t)ide analogs through the blood-brain barrier, which results in insufficient drug concentration to clear HBV (20). The presence of HBV in the CSF may increase the risk of CNS infection. Some theories proposed that molecular mimicry and immunological cross-reactivity between HBsAg and myelin antigens contribute to the development of demyelinating diseases in the CNS (21). Several hypotheses have also been proposed on how peripheral blood HBV can lead to CNS infection, including the disruption of the blood-brain barrier, production of hepatitis B immune complexes, and unequal transfer of virions and/or subviral particles across the blood-brain barrier (22–25). Though many previous studies have reported the detection of HBV DNA in the CSF of HBV-infected patients (20, 26–28), detecting HBV in the CSF of patients with NMOSD with concurrent HBV infection is rare, and the exact pathophysiological mechanisms are not clear (3, 6, 14). Our case suggests that HBV may trigger or contribute to the underlying mechanisms of NMOSD.

Limited studies have reported the replication of HBV in the CNS (20, 29–31). It remains uncertain whether HBV exclusively resides in the CNS following blood transmission or if it can actively replicate within the CNS of HBV-infected individuals. In our case, the high level of HBV replication might have resulted from blood contamination during the lumbar puncture procedure, while the absence of red blood cells in the CSF suggests that significant contamination is improbable. Moreover, Ene et al. isolated HBV from the CSF of patients co-infected with HIV and HBV and noticed that the replication potential of HBV in these patients could contribute to its ability to replicate in the CNS and trigger autoimmune inflammation (29).

Corticosteroids, a common first-line treatment for NMOSD, have the potential to reactivate HBV by suppressing T cell-mediated immunity and activating a glucocorticoid-responsive transcriptional element in the HBV genome (32). Antiviral treatment can mitigate both HBV integration and hepatocyte clonal expansion (33). Consistent with previous studies (3, 6, 14), our patient received high-dose methylprednisolone pulse therapy along with antiviral and hepatoprotective medications. Although some fluctuations in the results of liver function tests was noted, there was no evident elevation or hepatitis B outbreak. Researchers recommend prophylactic antiviral treatment for patients receiving high-dose methylprednisolone therapy (12, 34). Accordingly, our patient also received antiviral and hepatoprotective medications before initiating high-dose methylprednisolone pulse therapy.

In the remission phase, immunosuppressive agents are crucial to prevent relapse. It’s noteworthy that spontaneous HBVr may occur, but it is more commonly triggered by immunosuppressive therapy (12, 31). Currently, Eculizumab, inebilizumab, satralizumab, and ravulizumab are approved by the US FDA and the European Medicines Agency (EMA) for the treatment of AQP4-Ab positive NMOSD (35, 36). In China, ravulizumab is not approved for the treatment of NMOSD, whereas eculizumab and satralizumab are relatively expensive and not covered by medical insurance. Inebilizumab is a humanized anti-CD19 monoclonal antibody, while ofatumumab is a fully human anti-CD20 monoclonal antibody (36). Considering that CD19 is more widely expressed in B-lineage cells than CD20, we chose to use ofatumumab initially, which we thought might be safer (37). Although ofatumumab is an off-label drug, its mechanism of action suggests its potential use in the treatment of NMOSD. The efficiency of ofatumumab in patients with NMOSD has been reported in numerous studies (38–41). An observational study also found that patients with chronic lymphocytic leukemia combined with HBV did not experience HBVr after receiving ofatumumab treatment (42). Similarly, after ofatumumab treatment, not only did our patient’s symptoms significantly improve, but also HBVr did not occur. However, we later decided to switch the patient to inebilizumb, as it is the only medication covered by the medical insurance system for NMOSD in China. A recent report by Sadowsky, D. et al. described a case of an NMOSD patient with concurrent HBV and tuberculosis who switched from AZA to inebilizumab; however, neither the efficacy nor the tolerance of inebilizumab was reported (14). In our case, no HBV outbreaks, worsening liver function, or adverse events were observed at the last follow-up. We demonstrated the safety and efficacy of both ofatumumab and inebilizumab in NMOSD patients with HBV infection following the administration of anti-HBV agents and hepatoprotective drugs. However, this case report has certain limitations. Due to the insufficient evidence regarding the treatment of NMOSD patients with concurrent HBV infection and the use of ofatumumab and inebilizumab in such patients, the therapeutic regimen we adopted needs further research and observation. It remains unclear whether the patient’s condition will recur in the future and whether there will be adverse reactions.

In summary, our case suggests that ofatumumab and inebilizumab might serve as an effective and safe alternative for NMOSD patients with concurrent HBV infection. Large-scale studies are needed for further verification.

## Patient perspective

The patient conveyed that the entire course has been exceptionally challenging. Due to the initially presenting symptoms of nausea and vomiting and not receiving proper examination and medical consultation, she experienced the consequences of delayed diagnosis and management. Following diagnosis and initial treatment, her condition worsened, leading to frustration and emotional distress. Speech impairment, swallowing difficulties, choking on liquids, and limb weakness intensified her anxiety. However, after starting immunosuppressive therapy, she noticed a gradual improvement in her symptoms. The improvement in symptoms greatly encouraged her. Following the improvement in her breathing ability, she no longer required non-invasive ventilation, this also alleviated some of the financial burdens associated with medical care. The patient was highly satisfied with the treatment efficacy, drug safety, and the care received during hospitalization.

## Data availability statement

The original contributions presented in the study are included in the article/**Supplementary Material**. Further inquiries can be directed to the corresponding author.

## Ethics statement

The studies involving humans were approved by the Research Ethics Committee of the Medical School of Sichuan University. The studies were conducted in accordance with the local legislation and institutional requirements. Written informed consent for participation in this study was provided by the participants’ legal guardians/next of kin. Written informed consent was obtained from the individual(s) for the publication of any potentially identifiable images or data included in this article.

## Author contributions

LC: Conceptualization, Formal analysis, Methodology, Software, Supervision, Writing – original draft. XL: Conceptualization, Writing – review & editing. HZ: Conceptualization, Writing – review & editing. JL: Conceptualization, Writing – review & editing. DZ: Conceptualization, Supervision, Writing – review & editing. ZH: Conceptualization, Formal analysis, Funding acquisition, Project administration, Supervision, Writing – review & editing.

## Supplementary material

Supplementary Figure 1 The result of anti-aquaporin 4 antibody (Anti-AQP4) and anti-aquaporin 1 antibody (Anti-AQP1) detection in the serum and cerebrospinal fluid (CSF). Anti-AQP4 and Anti-AQP1 antibodies in the serum and CSF of the patient were positive, as tested by the cell-based assay (CBA) method. **(A)** Anti-AQP4 in serum; **(B)** Anti-AQP1 in serum; **(C)** Anti-AQP4 in CSF; **(D)** Anti-AQP1 in CSF.
