# Supplementary figures and images for: Case report: Identification of Hepatitis B Virus in the cerebrospinal fluid of neuromyelitis optica spectrum disorders and successful treatment with ofatumumab and inebilizumab
Source: Front Immunol. 2024 Feb 15;15:1351782. doi: 10.3389/fimmu.2024.1351782 (PMC10902669; doi:10.3389/fimmu.2024.1351782)

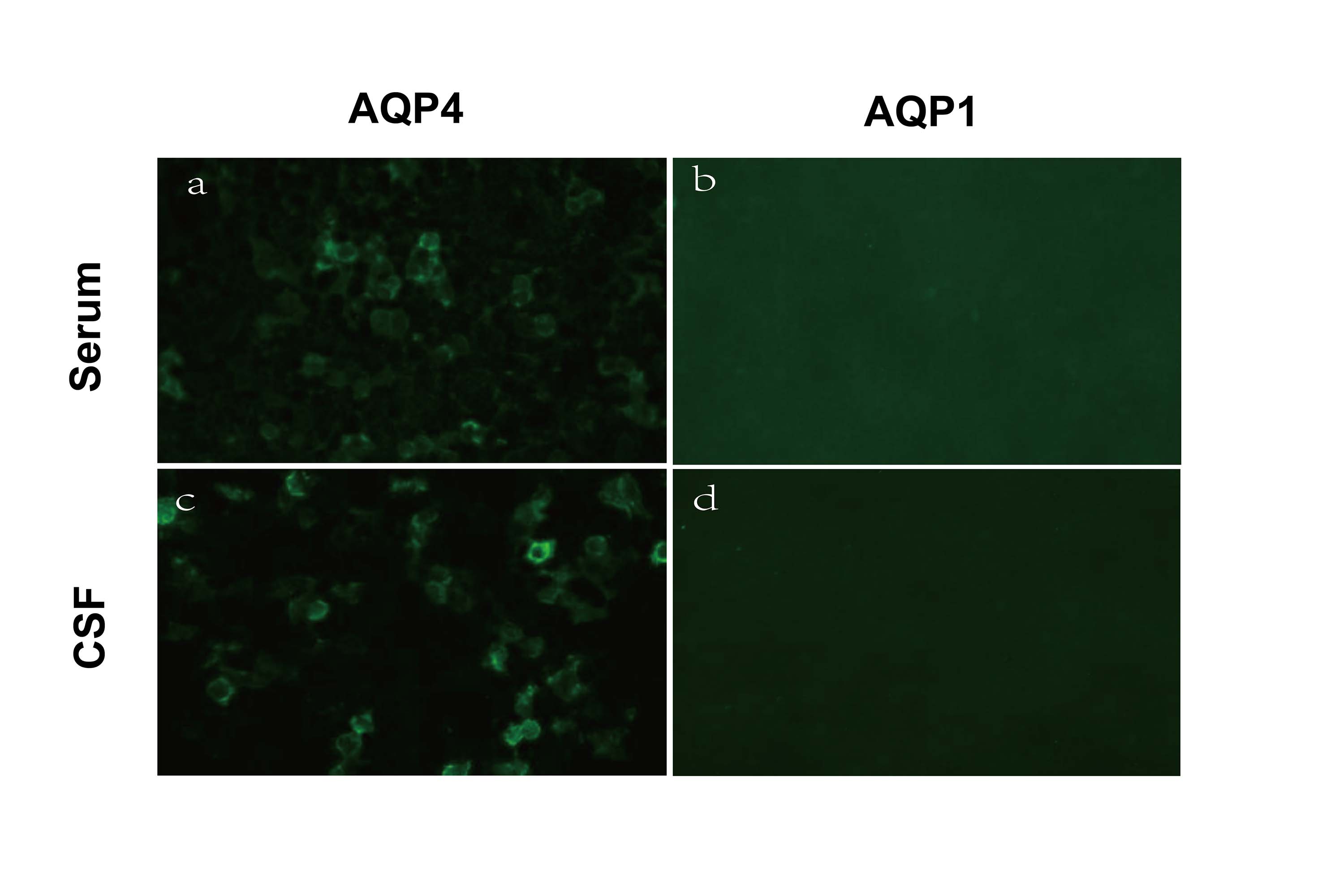

Supplement: Supplementary Figure 1 — The result of anti-aquaporin 4 antibody (Anti-AQP4) and anti-aquaporin 1 antibody (Anti-AQP1) detection in the serum and cerebrospinal fluid (CSF). Anti-AQP4 and Anti-AQP1 antibodies in the serum and CSF of the patient were positive, as tested by the cell-based assay (CBA) method. (A) Anti-AQP4 in serum; (B) Anti-AQP1 in serum; (C) Anti-AQP4 in CSF; (D) Anti-AQP1 in CSF. [file Image_1.jpeg]
